# Anterior abdominal wall cysticercosis-the role of high-resolution USG

**DOI:** 10.4103/0971-3026.41844

**Published:** 2008-08

**Authors:** Amit Mittal, Sanjeev Gupta, Vinod Mehta, Rakesh Gupta

**Affiliations:** Department of Radiodiagnosis, MM Institute of Medical Sciences and Research, Mullana, Ambala, India. E-mail: amitmittalrad@yahoo.co.in; 1 Department of Dermatology and Venereology, MM Institute of Medical Sciences and Research, Mullana, Ambala, India

Dear Sir,

We have read the article on high-resolution USG of the anterior abdominal wall by Sudheer Gokhale.[1] He has given clear descriptions of almost all the pathologies of the anterior abdominal wall as seen on high-resolution USG. We want to draw attention to another very important pathology of the anterior abdominal wall that is endemic in our country, which is subcutaneous or intramuscular cysticercosis. High-resolution USG is diagnostic for subcutaneous and intramuscular cysticercosis.[2,3] We have come across two patients with anterior abdominal wall cysticercosis.

**Figure 1:**
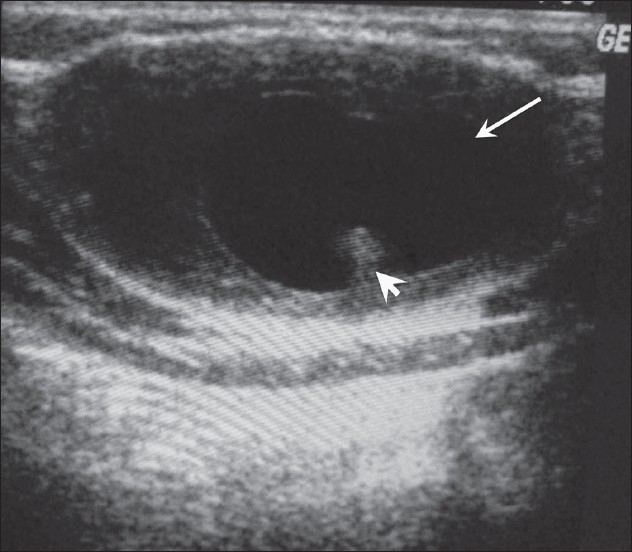
USG shows a well-defined cyst (arrow) with an echogenic scolex (arrowhead), in the subcutaneous tissues

The first patient, a 28-year-old man, presented with a swelling on the left side of the anterior abdominal wall that had been present for 1 month. Clinically, it was diagnosed as a lipoma or neurofibroma. The patient was sent for a high-resolution USG study, which revealed a well-defined cystic lesion with an echogenic nidus in the subcutaneous tissue in the area of the swelling [Figure 1]. Based on these findings, the diagnosis of subcutaneous cysticercosis was made.

**Figure 2:**
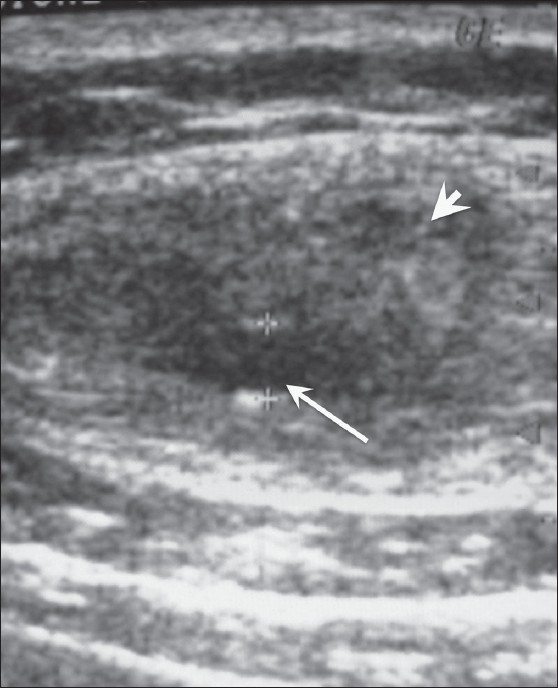
USG shows a cystic lesion (arrow) with a hypoechoic area (arrowhead) in the left rectus abdominis muscle

The second patient was a 35-year-old man presenting with a painful swelling on the right side of the anterior abdominal wall for 8-10 days. On examination, the swelling was tender and hard and the skin overlying the swelling was inflamed. There was no history of fever or trauma. Clinically, it was diagnosed as an abscess. On high-resolution USG, there was a small cyst of size 3 mm, with a surrounding 15 × 22-mm hypoechoic area in the left rectus abdominis muscle [Figure 2]. Based on these findings, a diagnosis of intramuscular cysticercosis with surrounding inflammatory phlegmon in the rectus abdominis muscle was made.

In both these cases no further investigations were done. The patients recovered fully after treatment with albendazole and corticosteroids.

High-resolution USG plays an important role in establishing the diagnosis of muscular and subcutaneous cysticercosis. The salient diagnostic feature of a cysticercus granuloma is the presence of an oval or rounded well-defined cystic lesion, with an eccentric echogenic nidus within. If this picture is seen in a subcutaneous or intramuscular swelling, the diagnosis of cysticercosis can be made with great confidence and no further investigations are required;[2,3] biopsy or the less reliable fine needle aspiration cytology (FNAC) can be avoided. These patients can be managed conservatively and the diagnosis can be confirmed by the therapeutic response.[4]
